# Persistence of SARS-CoV-2 N-Antibody Response in Healthcare Workers, London, UK

**DOI:** 10.3201/eid2704.204554

**Published:** 2021-04

**Authors:** Madhumita Shrotri, Ross J. Harris, Alison Rodger, Timothy Planche, Frances Sanderson, Tabitha Mahungu, Alastair McGregor, Paul T. Heath, The LondonCOVID Group, Colin S. Brown, Jake Dunning, Susan Hopkins, Shamez Ladhani, Meera Chand

**Affiliations:** Public Health England, London, UK (M. Shrotri, R.J. Harris, C.S. Brown, J. Dunning, S. Hopkins, S. Ladhani, M. Chand);; University College London, London (A. Rodger);; Royal Free NHS Foundation Trust, London (A. Rodger, T. Mahungu, C.S. Brown);; St. George’s University Hospitals NHS Foundation Trust, London (T. Planche);; St. George’s University of London (T. Planche, P.T. Heath, S. Ladhani);; Imperial College Healthcare NHS Trust, London (F. Sanderson);; London North West University Healthcare NHS Trust, London (A. McGregor)

**Keywords:** COVID-19, severe acute respiratory syndrome coronavirus 2, serology, antibodies, epidemiology, health personnel, healthcare workers, coronavirus disease, SARS-CoV-2, viruses, respiratory infections, zoonoses, London

## Abstract

Prospective serosurveillance of severe acute respiratory syndrome coronavirus 2 in 1,069 healthcare workers in London, UK, demonstrated that nucleocapsid antibody titers were stable and sustained for <12 weeks in 312 seropositive participants. This finding was consistent across demographic and clinical variables and contrasts with reports of short-term antibody waning.

The durability of antibody responses to severe acute respiratory syndrome coronavirus 2 (SARS-CoV-2), the virus responsible for coronavirus disease (COVID-19), is of scientific and strategic interest for public health systems worldwide. After SARS-CoV-2 infection, antibodies are produced against multiple viral epitopes, including the nucleocapsid (N) protein, which is highly immunogenic and abundantly expressed (*1*). A key concern is the potential for rapid waning of antibodies and seroreversion (loss of detectable antibodies), as seen with other novel betacoronaviruses (*2*), which might represent declining immunity and could compromise serosurveillance. 

Frontline healthcare workers are a vital population for serosurveillance because they are at greater risk than the general population. We describe findings from a serosurveillance study conducted in London, UK, by Public Health England (PHE).

## The Study

We conducted prospective serosurveillance of healthcare professionals in secondary care settings across London beginning March 30, 2020. Healthcare workers were recruited by hospital research teams and provided written informed consent. Demographic, occupational, and clinical data were collected at baseline, including self-reported previous laboratory-confirmed COVID-19. Participants provided blood samples and completed symptom surveys at baseline and 2-weekly intervals until July 21, 2020, reporting any new illness or COVID-19 diagnosis. Blood samples were centrifuged and frozen locally; PHE then tested serum samples by using the Elecsys Anti-SARS-CoV-2 total antibody assay (Roche, https://www.roche.com), according to the manufacturer’s instructions. This test is an electrochemiluminescence immunoassay for antibodies targeting the N protein (IgG, IgM, or IgA) and produces a numeric cutoff index derived from comparison of the sample and calibrator signals (*3*). The surveillance protocol was approved by the PHE Research Ethics Governance Group (R&D REGG Ref: NR0192, March 31, 2020).

We compared differences in seropositivity between groups by using χ^2^ tests and multivariable logistic regression to provide adjusted odds ratios (aORs). We estimated biweekly seroconversion and seroreversion rates and binomial 95% CIs. We analyzed trends in individual-level antibody responses beginning 4 weeks after the first positive antibody test, which allowed time for responses to stabilize. We used mixed effects regression to analyze trends in log antibody titers and assessed fixed effects for differences in antibody response through likelihood ratio tests.

Surveillance involved 1,069 participants from 4 hospitals: Charing Cross (n = 192), Northwick Park (n = 217), Royal Free (n = 126), and St. George’s (n = 534). Of these, 850 participants had >4 sampling visits and 395 >6 sampling visits (over 10–12 weeks of follow-up). Overall, 312 (29%) participants had >1 positive antibody test (95% CI 26%–32%); of those, 181 (58%) had >8 weeks and 42 (13%) 12 weeks of follow-up after the first positive test (Appendix Table 1). Seropositivity varied between hospitals (p = 0.042), from 25% to 35%. In total, 109 (10.2%) participants self-reported laboratory-confirmed COVID-19, 407 (32%) reported respiratory illness, 5 (0.47%) reported hospitalization, and 794 (61%) did not report illness.

We observed no difference in seropositivity by sex, profession, performance of aerosol-generating procedures, employment in the emergency department, or immunocompromised status (Appendix Table 2). Participants 25–34 years of age had higher odds of seropositivity than those 35–44 years of age (aOR 1.57, 95% CI 1.09–2.26), but little difference was seen among older age groups. Those working in intensive care units had lower odds of seropositivity than participants from other hospital departments (aOR 0.58, 95% CI 0.38–0.91).

Most seropositive participants tested positive at baseline (279/312, 89%). Only 33 participants seroconverted during follow-up, corresponding to a biweekly rate of 1.2% (95% CI 0.8%–1.7%). We observed 4 seroreversions, corresponding to a biweekly rate of 0.4% (95% CI 0.1%–0.9%).

log antibody titers remained stable over time in seropositive participants, and little within-individual variability was observed (Figure). The general trend across all subgroups was a slight increase over time, although data are sparse for some groups.

**Figure:**
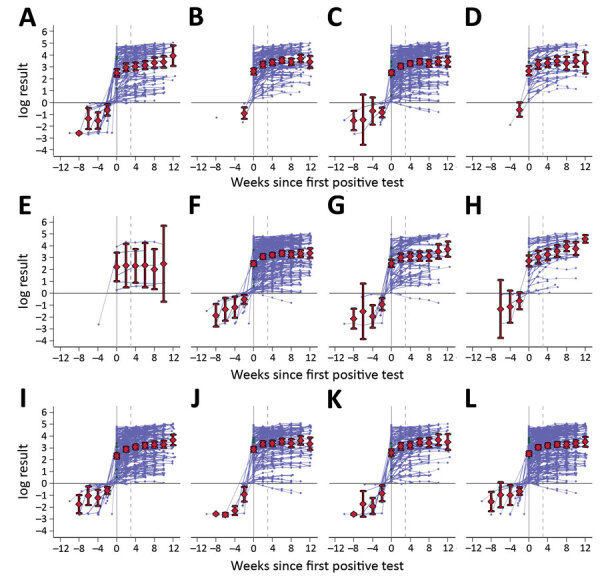
log antibody titers over time in participants with >1 positive test result by subgroups in study of nucleocapsid-antibody response in healthcare workers, London, UK. Subgroups are as follows: A) no self-reported illness (n = 99), B) coronavirus disease (COVID-19) diagnosis (n = 94), C) respiratory illness (n = 175), D) other illness (n = 43), E) immunocompromised (n = 6), F) general hospital employee (n = 204), G) emergency department employee (n = 71), H) intensive care unit employee (n = 38), I) age <40 years (n = 185), J) age >40 years (n = 127), K) male sex (n = 95), L) female sex (n = 217). Times are with respect to the date of the first positive test (week 0), and week 4 is indicated by dashed lines; previous negative results are also included. Individual responses are indicated by blue lines; mean titers with 95% CI for the mean are shown in red.

We modeled trends beginning 4 weeks after the first positive antibody test. The mean weekly change was a 3.9% increase (95% CI 3.2%–4.6%). The model enables individual variability and thus estimates a distribution in trends, which ranged from a 0.5% decrease to an 8.5% increase per week, at 1 SD below/above the mean.

Baseline response or subsequent trend did not differ by work setting, clinical symptoms, or laboratory-confirmed COVID-19; minimum likelihood ratio p value was 0.46. Participants >40 years of age had 30% higher antibody titers at baseline (p = 0.08) but less increase over time; weekly increase was 2.9% (95% CI 1.8%–4.0%) compared with 4.5% (95% CI 3.6%–5.4%) in those <40 years of age (p = 0.028). We observed similar baseline titers between women and men (p = 0.61) but different trends; women demonstrated a weekly increase of 3.4% (95% CI 2.6%–4.2%) compared with 5.2% (95% CI 3.8%–6.6%) in men (p = 0.035).

## Conclusions

In this study, N-antibody seropositivity was 29% among healthcare workers, and a small, sustained rise in antibody titers occurred over 12 weeks. The increase could be explained by the natural boosting of antibodies through repeated SARS-CoV-2 exposure; however, we saw no evidence of sporadic, sharp increases in antibodies in seropositive participants, and we observed little deviation from an overall linear trend. High initial seroprevalence and low subsequent seroconversion rates (Appendix Figures 1, 2) indicate that most exposures occurred before surveillance began. The low seroincidence after April might be attributable to changes in hospital infection control practices and national lockdown.

These findings demonstrate the short-term stability of N-antibody titers in healthcare staff, regardless of demographic or clinical differences. Seropositive participants not reporting any COVID-19 diagnosis or previous illness (even mild or atypical symptoms) demonstrated the same antibody trends as those who reported symptoms or laboratory-confirmed COVID-19, thereby supporting N-antibody testing as a reliable surveillance indicator. Although seroreversion was uncommon, such rates, if sustained, might be concerning in the long term.

Although cross-reactivity against the N protein has been observed and appears more prevalent than cross-reactivity against the spike (S) protein (E.M. Anderson, unpub. data, https://doi.org/10.1101/2020.11.06.20227215; C.F. Houlihan, unpub. data, https://doi.org/10.1101/2020.06.08.20120584), the risk for false positives because of preexisting human coronavirus antibodies seems low on the basis of available data. The Elecsys assay demonstrated >99.5% specificity in 2 independent evaluations using large numbers of prepandemic control samples (*3*,*4*) and demonstrated high positive predictive value at an estimated 10% seroprevalence. Nonetheless, this study is limited by use of a single immunoassay, by self-reported data on COVID-19 diagnosis, and by limited testing early in the pandemic.

Several studies have demonstrated substantial declines in antibody titers over 3–5 months by using anti-S or anti–receptor-binding domain immunoassays (*5*–*9*). Although findings are not consistent across all reports (*6*,*10*), disparities could be explained by shorter follow-up periods that missed later decline. In contrast, the few studies conducting serial testing for >3 months by using N-antibody assays, particularly the Elecsys assay, report that titers remained steady (*9*) or increased (*11*; F. Muecksch, unpub. data, https://doi.org/10.1101/2020.08.05.20169128). These studies were limited by small sample sizes, single-site recruitment, and few time points with long sampling intervals. Our study replicates these findings in a large, multicenter cohort with frequent sampling and focuses on healthcare workers with mostly asymptomatic or mild disease, with robust statistical analysis to demonstrate consistent findings across all groups. These data can usefully inform serosurveillance strategies during the second wave.

For unknown reasons, N-antibodies appear highly stable in the short term, despite demonstrating no functional role; whether this stability would persist over longer follow-up periods remains to be answered. Although less useful as correlates of immunity, N-antibodies could serve a critical role in serosurveillance as S-based vaccines are deployed, helping to distinguish infection-induced seroconversion from vaccine-induced seroconversion.

Appendix Additional information about persistence of SARS-CoV-2 N-antibody response in healthcare workers, London, UK.

## References

[1] Liu W, Liu L, Kou G, Zheng Y, Ding Y, Ni W, et al. Evaluation of nucleocapsid and spike protein-based enzyme-linked immunosorbent assays for detecting antibodies against SARS-CoV-2. J Clin Microbiol. 2020;58:1–7. 10.1128/JCM.00461-20 32229605 PMC7269413

[2] Kellam P, Barclay W. The dynamics of humoral immune responses following SARS-CoV-2 infection and the potential for reinfection. J Gen Virol. 2020;101:791–7. 10.1099/jgv.0.001439 32430094 PMC7641391

[3] Public Health England. Evaluation of Roche Elecsys Anti-SARS-CoV-2 serology assay for the detection of anti-SARS-CoV-2 antibodies. London: Public Health England; 2020 [cited 2020 Dec 21]. https://assets.publishing.service.gov.uk/government/uploads/system/uploads/attachment_data/file/891598/Evaluation_of_Roche_Elecsys_anti_SARS_CoV_2_PHE_200610_v8.1_FINAL.pdf

[4] Ainsworth M, Andersson M, Auckland K, Baillie JK, Barnes E, Beer S, et al.; National SARS-CoV-2 Serology Assay Evaluation Group. Performance characteristics of five immunoassays for SARS-CoV-2: a head-to-head benchmark comparison. Lancet Infect Dis. 2020;20:1390–400. 10.1016/S1473-3099(20)30634-4 32979318 PMC7511171

[5] Ibarrondo FJ, Fulcher JA, Goodman-Meza D, Elliott J, Hofmann C, Hausner MA, et al. Rapid decay of anti–SARS-CoV-2 antibodies in persons with mild Covid-19. N Engl J Med. 2020;383:1085–7. 10.1056/NEJMc2025179 32706954 PMC7397184

[6] Bölke E, Matuschek C, Fischer JC. Loss of Anti-SARS-CoV-2 Antibodies in Mild Covid-19. N Engl J Med. 2020;383:1694–5. 10.1056/NEJMc2027051 32966710

[7] Wajnberg A, Amanat F, Firpo A, Altman DR, Bailey MJ, Mansour M, et al. Robust neutralizing antibodies to SARS-CoV-2 infection persist for months. Science. 2020;370:1227–30. 10.1126/science.abd7728 33115920 PMC7810037

[8] Perreault J, Tremblay T, Fournier MJ, Drouin M, Beaudoin-Bussières G, Prévost J, et al. Waning of SARS-CoV-2 RBD antibodies in longitudinal convalescent plasma samples within 4 months after symptom onset. Blood. 2020;136:2588–91. 10.1182/blood.2020008367 33001206 PMC7714093

[9] Seow J, Graham C, Merrick B, Acors S, Pickering S, Steel KJA, et al. Longitudinal observation and decline of neutralizing antibody responses in the three months following SARS-CoV-2 infection in humans. Nat Microbiol. 2020;5:1598–607. 10.1038/s41564-020-00813-8 33106674 PMC7610833

[10] Wang Y, Zhang L, Sang L, Ye F, Ruan S, Zhong B, et al. Kinetics of viral load and antibody response in relation to COVID-19 severity. J Clin Invest. 2020;130:5235–44. 10.1172/JCI138759 32634129 PMC7524490

[11] Fill Malfertheiner S, Brandstetter S, Roth S, Harner S, Buntrock-Döpke H, Toncheva AA, et al. Immune response to SARS-CoV-2 in health care workers following a COVID-19 outbreak: A prospective longitudinal study. J Clin Virol. 2020;130:104575. 10.1016/j.jcv.2020.104575 32805631 PMC7406471

