# Supplementary material for: Persistence of SARS-CoV-2 N-Antibody Response in Healthcare Workers, London, UK
Source: Emerg Infect Dis. 2021 Apr;27(4):1155–8. doi: 10.3201/eid2704.204554 (PMC8007325; doi:10.3201/eid2704.204554)
Supplement: Appendix — Additional information about persistence of SARS-CoV-2 N-antibody response in healthcare workers, London, UK. [file 20-4554-Techapp-s1.pdf]

Article DOI: https://doi.org/10.3201/eid2704.204554

Persistence of SARS-CoV-2 N-Antibody Response in Healthcare Workers, London, UK

Appendix

Appendix Table 1. Numbers and percentages of seropositive participants (n = 312) by length of follow-up after first positive antibody test in study of N-antibody response in healthcare workers, London, UK

Weeks of follow-up after first positive test	Frequency	Percentage	Cumulative percentage
0	18	5.77	5.77
2	12	3.85	9.62
4	22	7.05	16.67
6	79	25.32	41.99
8	51	16.35	58.33
10	88	28.21	86.54
12	42	13.46	100.00
Total	312	100.00	100.0

Appendix Table 2. Results from multivariable logistic regression examining association of various demographic, occupational, and clinical factors on the odds of seropositivity in study of SARS-CoV-2 N-antibody response in healthcare workers, London, UK*

Variable	aOR of seropositivity (95% CI)	p value
Age group, y		
15–24	1.41 (0.70–2.84)	0.331
25–34	1.57 (1.09–2.26)	0.016
35–44	1	
45–54	1.26 (0.82–1.96)	0.294
≥55	0.93 (0.47–1.84)	0.843
Sex		
M	1	
F	0.89 (0.64–1.24)	0.497
Professional role		
Doctor	1	
Nurse	0.92 (0.65–1.30)	0.633
Other	1.21 (0.82–1.77)	0.341
Emergency department setting	0.95 (0.66–1.36)	0.761
ICU setting	0.58 (0.38–0.91)	0.016
Aerosol-generating procedures	1.08 (0.78–1.48)	0.655
Immunocompromised	1.19 (0.44–3.24)	0.736

*aOR, adjusted odds ratio; ICU, intensive care unit.

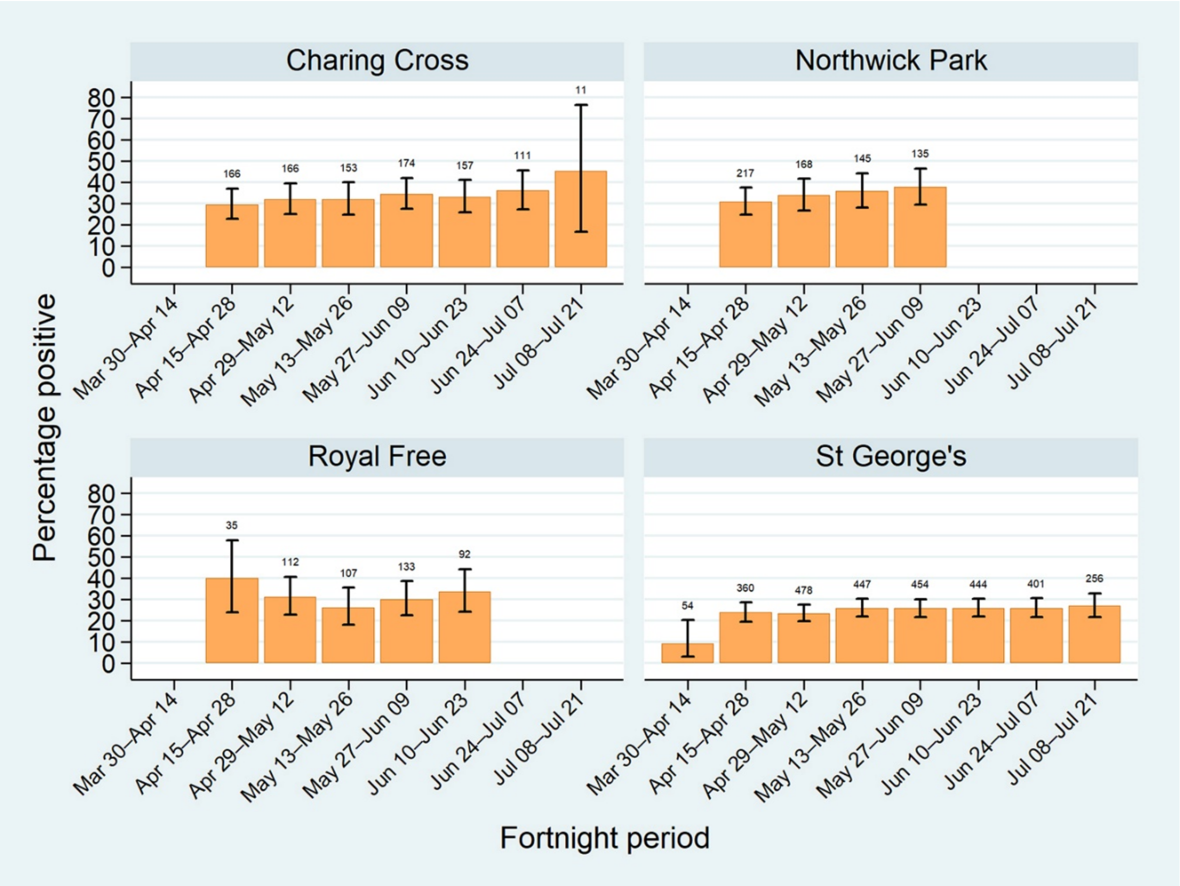

Appendix Figure 1. Percentage of positive samples by study site and 2-week periods with binomial 95% CI in study of severe acute respiratory syndrome coronavirus 2 N-antibody response in healthcare workers, London, UK. Sample sizes are indicated above each bar.

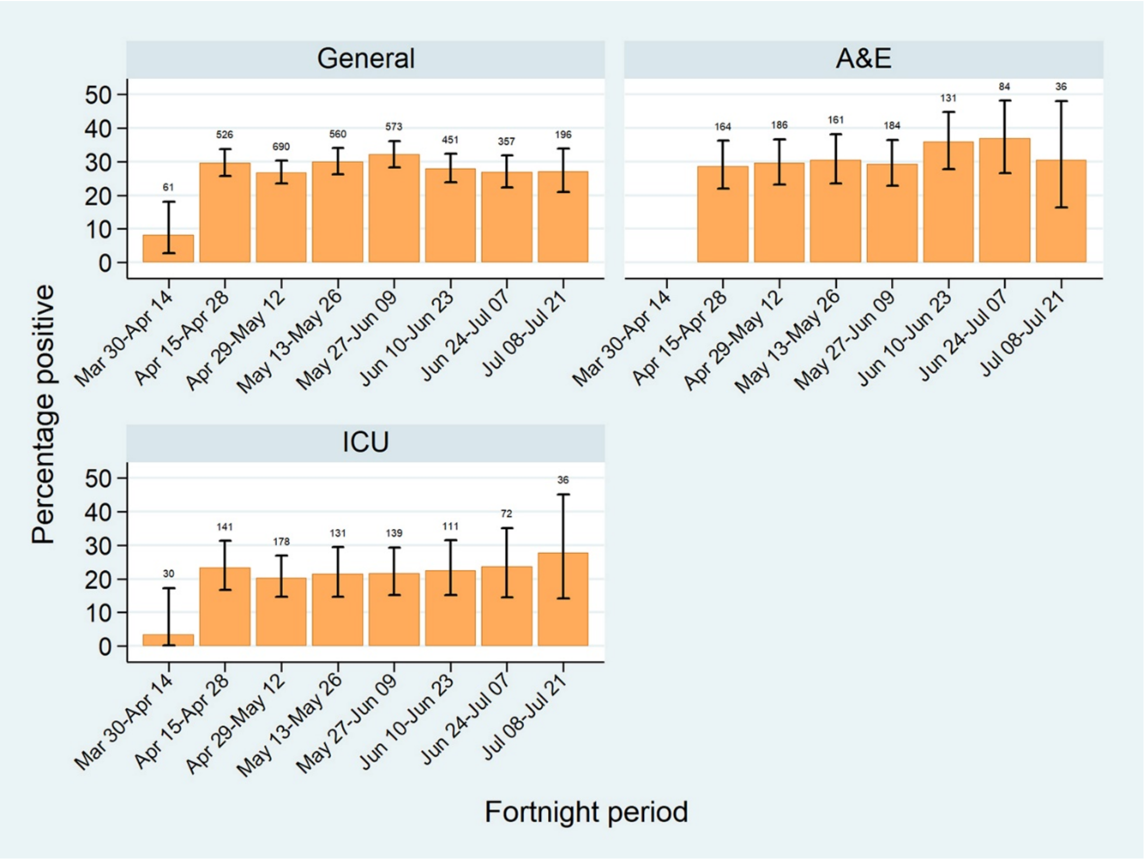

Appendix Figure 2. Percentage of positive samples by hospital department and 2-week periods with binomial 95% CI in study of severe acute respiratory syndrome coronavirus 2 N-antibody response in healthcare workers, London, UK. Sample sizes are indicated above each bar. ICU, intensive care unit.
